# The reduction of adult neurogenesis in depression impairs the retrieval of new as well as remote episodic memory

**DOI:** 10.1371/journal.pone.0198406

**Published:** 2018-06-07

**Authors:** Jing Fang, Selver Demic, Sen Cheng

**Affiliations:** 1 Institute for Neural Computation, Ruhr University Bochum, Bochum, Germany; 2 Mercator Research Group “Structure of Memory”, Ruhr University Bochum, Bochum, Germany; 3 Faculty of Psychology, Ruhr University Bochum, Bochum, Germany; 4 St. Elisabeth Hospital, Gütersloh, Germany; Radboud University Medical Centre, NETHERLANDS

## Abstract

Major depressive disorder (MDD) is associated with an impairment of episodic memory, but the mechanisms underlying this deficit remain unclear. Animal models of MDD find impaired adult neurogenesis (AN) in the dentate gyrus (DG), and AN in DG has been suggested to play a critical role in reducing the interference between overlapping memories through pattern separation. Here, we study the effect of reduced AN in MDD on the accuracy of episodic memory using computational modeling. We focus on how memory is affected when periods with a normal rate of AN (asymptomatic states) alternate with periods with a low rate (depressive episodes), which has never been studied before. Also, unlike previous models of adult neurogenesis, which consider memories as static patterns, we model episodic memory as sequences of neural activity patterns. In our model, AN adds additional random components to the memory patterns, which results in the decorrelation of similar patterns. Consistent with previous studies, higher rates of AN lead to higher memory accuracy in our model, which implies that memories stored in the depressive state are impaired. Intriguingly, our model makes the novel prediction that memories stored in an earlier asymptomatic state are also impaired by a later depressive episode. This retrograde effect exacerbates with increased duration of the depressive episode. Finally, pattern separation at the sensory processing stage does not improve, but rather worsens, the accuracy of episodic memory retrieval, suggesting an explanation for why AN is found in brain areas serving memory rather than sensory function. In conclusion, while cognitive retrieval biases might contribute to episodic memory deficits in MDD, our model suggests a mechanistic explanation that affects all episodic memories, regardless of emotional relevance.

## Introduction

Major depressive disorder (MDD) is the most common mood disorder, estimated to affect 20% of the population at some point of a person’s lifetime [1–3]. MDD is characterized by a constellation of behavioural, emotional and cognitive symptoms, especially in the domain of memory [4]. Numerous studies have reported a selective impairment of episodic memory during depressive episodes [5–8]. Some studies even find an almost linear relationship between scores on a depression rating scale and episodic memory performance [9, 10]. Unlike episodic memory, however, semantic memory, the other type of declarative memory, is relatively intact in MDD patients [11, 12].

The mechanisms underlying MDD are not understood. The neurogenic theory of depression suggests that impaired adult neurogenesis (AN) in the dentate gyrus (DG) triggers depression and that restoration of AN leads to recovery [13]. AN refers to the process that generates new neurons beyond development in adulthood. It occurs in only two regions of the mammalian brain, one of which is the DG. A number of experimental studies have observed a reduction of AN in animal models of MDD [14–18]. While there are no direct measurements of AN in brains of MDD patients, both post-mortem [19] and high-resolution MRI volumetric [20, 21] studies consistently find smaller DG sizes in subjects who had suffered or were suffering from MDD. In addition, animal studies indicate that the rate of AN can be increased by antidepressant treatment [22–24] and ablating AN suppresses the antidepressant effect of the drug [14, 25]. However, the clear picture painted by these studies is complicated by findings that even a complete reduction of AN [25] does not produce the behavioural symptoms of MDD, see [26] for a review. Nevertheless, even though the role of AN in the etiology of MDD remains uncertain, the evidence strongly suggests that there is a correlation between MDD and AN in DG.

The DG is a subregion of the hippocampus, which is heavily involved in the storage and retrieval of episodic memory [27–29]. Marr [30] suggested that memories are stored in an associative network that is implemented in the recurrent connections of hippocampal CA3. Computational studies suggest that memory patterns in CA3 have to be uncorrelated to avoid interference between memories. Since sensory inputs are highly correlated, the hippocampal network has to pre-proccess these input patterns to reduce the correlations before they can be stored in CA3 [31]. This process is called pattern separation and the DG, which receives inputs from the entorhinal cortex (EC) and sends direct projections to CA3, has been suggested to be especially suitable for this purpose [30, 32–34]. There is mounting empirical support for the hypothesis that AN in DG plays a role in minimizing interference between overlapping memories. Animals with AN impairment show a deficit in spatial discrimination [35–37] and in learning overlapping odour pair discriminations [38]. On the other hand, increasing AN improves pattern separation [39]. An fMRI study in humans also shows that the presentation of objects that are very similar, but not identical, to previously learned objects increases BOLD activity in human DG/CA3 [40]. Linking MDD, AN, and pattern separation together, recent studies in humans found a negative correlation between depression scores and pattern separation performance [41, 42]. Déry et al. also find that the memory deficit in depression is selective for a neurogenesis-dependent task, and does not occur in other hippocampus-dependent control tasks [41].

In contrast to the abundance of experimental and clinical studies on the link between MDD and cognitive deficits, there are few modelling studies on this topic. One example is the study by Becker et al., who proposed a functional cluster hypothesis in their theoretical model by which cells born at a particular time in the DG encode a context that binds together all memories formed in that context [43]. An AN deficit then causes deficits in contextual memory. By contrast, the vast majority of computational studies focus on the broader question of how AN contributes to normal learning and memory, see [44] for a review. AN is implemented either by replacing trained cells with new naïve cells or generate additional new cells. In simple feedforward architectures, neural replacement improves the encoding of new memories at the cost of losing previously stored memories [45–48]. By contrast, adding new neurons to the network can help avoid catastrophic interference [49] and can preserve old memories as well as store and retrieve new memories [50]. Aimone et al. emphasizes the role of newborn immature granule cells which are more broadly tuned to a wide range of inputs [51]. Their model suggests that immature neurons increase the similarity between contemporaneous events, but once they are mature, they separate events that occur in different time periods. Nonetheless, these computational studies do not account for the specific episodic memory deficits in MDD.

Finally, little is known about how dynamic changes in the rate of AN might affect episodic memories. The time course of MDD is highly dynamic [52, 53] and involves transitions between depressive episodes, when the rate of AN is putatively low, and asymptomatic states, when the rate of AN is putatively higher. Although memory deficits in depressive patients have been reported in various episodic memory tasks, these studies generally examine the memories both formed and retrieved in the depressive state. The accuracy of memories formed during asymptomatic states and retrieved during depressive episodes, or vice versa, has not been studied using controlled experiments. Note that this cannot be achieved by asking depressive patients to recall auto-biographical memories stored in a previous asymptomatic state, since not all auto-biographical memory can be considered episodic memory [28, 54].

In this study, we develop a computational model that accounts for episodic memory deficits in MDD by assuming that MDD leads to a reduction in DG AN, which in turn leads an impairment in pattern separation, which eventually impairs episodic memory retrieval. Unlike previous models of adult neurogenesis, which consider memories as static patterns, we model episodic memory as sequences of neural activity patterns. Also, we examine for the first time how episodic memories are affected by the dynamics of MDD. To simulate this dynamics, the model differentiates an asymptomatic state with a normal rate of AN and a depressive state with reduced rate of AN. We compare the retrieval of memories stored and retrieved in the same state as well as memories stored in one state and retrieved in another. We find that pattern separation indeed improves episodic memory retrieval as well as its robustness to the retrieval noise. Retrieval performance is significantly worse for memories stored and retrieved in the depressive state as compared to the asymptomatic state. Interestingly, our model predicts an retrograde effect of MDD on memories formed in an earlier asymptomatic state. This effect is a novel prediction of our model, which has not been previously reported by any study, experimental or computational.

## Methods

### Memory model

**Fig 1:**
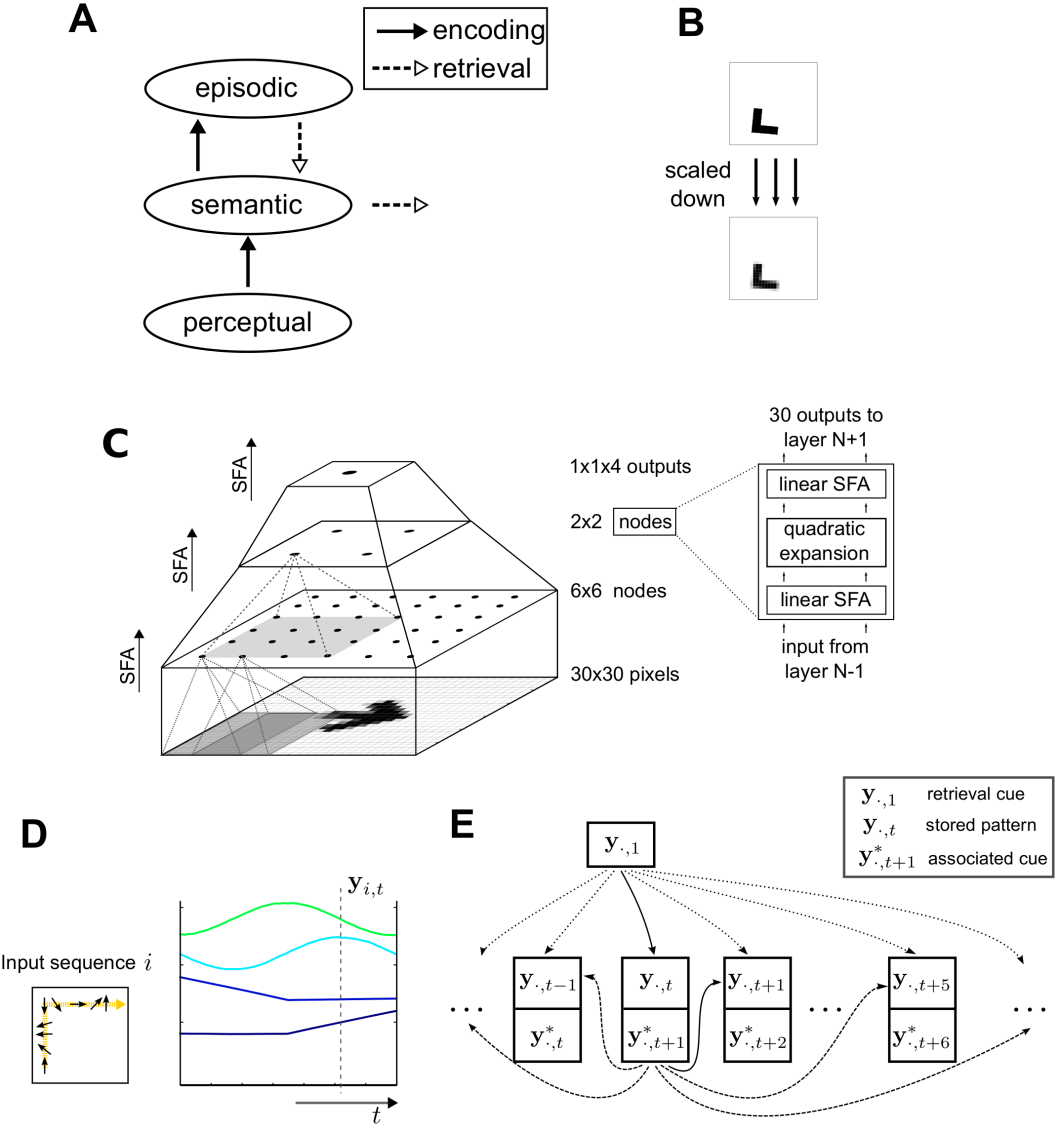
Schematic of the episodic memory model. A. The relationship between systems involved episodic memory. B. Example of the input stimuli. Top: 300 × 300 black-and-white pixels; bottom: pattern scaled down to 30 × 30 greyscale pixels. C. Hierarchical network of slow feature analysis (SFA) as a model of the semantic system. The dots in each layer symbolize SFA nodes. The grey patches indicate the receptive field of each node, partially overlapping with the neighbouring nodes’ receptive fields. As an ensemble nodes in a given layer cover the full input space. Each node performs a number of processing steps as visualized on the right hand side. The activity in the top layer is taken as the output of the semantic system in our memory model. D. Example of the output of the semantic representation layer. The object in the input sequence *i* moves along the trajectory (yellow arrow) and rotates by 360 degrees (indicated by black arrows). Shown on the right are the four slowest features calculated by the SFA-network. The feature values at time *t*, **y**_*i*,*t*_ (dashed line), form a semantic (more abstract) representation of the input. E. Sequence storage network (see main text in Methods for details).

To study episodic memory storage and retrieval, we adopted a model that we proposed and studied in earlier work [55]. The model consists of three systems (the perceptual, semantic and episodic system), which are arranged hierarchically (Fig 1A). This model assumes that episodic memories are represented as sequences of activation patterns, which are stored in the hippocampus [28, 29, 56]. These activation patterns are the outputs of a semantic representation network in neocortex, which generates low dimensional semantic representations of high dimensional sensory input. In other word, episodic memory in the model is defined as sequences of semantic representations.

#### Input stimuli

The inputs consisted of sequences of 2-d images containing an L-shaped object (300 × 300 binary pixels, Fig 1B). To keep the memory requirement and run-time at a manageable levels, the patterns were scaled down to 30 × 30 greyscale images by averaging across 10 × 10 patches before they are process by the semantic network. The training sequences were generated in such a way to ensure that the semantic representations that emerged during training (see below) can be readily interpreted. In the sequences, the object’s center (*r*_1_, *r*_2_) moved along a Lissajous curve:

$$\begin{array}{c} r_{i}\left(t\right)=a_{i}\sin\left(b_{i}t+c_{i}\right), \end{array}$$ (1)

where *i* = 1, 2 and *a*_1_ = *a*_2_ = 100 due to the square input space. The ratio $\frac{b_{1}}{b_{2}}=\frac{\pi }{3}$ was set to an irrational number, so that theoretically the trajectory would never repeat. The rotation of the object was described by

$$\begin{array}{c} \varphi \left(t\right)=\omega t+\varphi_{0}, \end{array}$$ (2)

where *φ* is the orientation and *ω* = 0.025*e*.

Test sequences were generated using a different statistics to ensure that our results are not selective to the specific input statistics used during training. In the test sequences, the object moved along a random walk trajectory, where the object can translate horizontally and vertically in each time step. The step sizes in the two directions were drawn independently from a normal distribution *v* ∼ *N*(5, 2.2). If a step would have taken the object beyond the boundary, the object was reflected on the boundary instead. The rotation of the object also followed a random walk, where the steps are drawn from *δ*_*φ*_ ∼ *N*(0, (0.035*e*)^2^).

#### Semantic representation network

A feedforward hierarchical structure based on slow feature analysis (SFA) algorithm was implemented as the semantic representation layer (Fig 1C). SFA finds instantaneous scalar input-output functions that generate slowly varying output signals from quickly varying inputs [57]. Specifically, in a given function space *F* and given a multidimensional input signal **x**(*t*), SFA finds a set of functions {*g*^(1)^(**x**), *g*^(2)^(**x**), ⋯, *g*^(*i*)^(**x**), ⋯}, where *g*^(*i*)^(**x**) ∈ *F*, such that the output signals {*y*^(1)^(*t*), *y*^(2)^(*t*), ⋯, *y*^(*i*)^(*t*), ⋯}, where *y*^(*i*)^(*t*) ≔ *g*^(*i*)^(**x**(*t*)), vary slowly. To this end, the Delta value

$$\begin{array}{c} \Delta \left(y^{\left(i\right)}\right):={\left\langle {\left({\dot{y}}^{\left(i\right)}\right)}^{2}\right\rangle }_{t} \end{array}$$ (3)

is minimized, under the following constraints:

$$\begin{array}{cc} {\left\langle y^{\left(i\right)}\right\rangle }_{t}=0\; & \text{(zero}\;\text{mean),} \end{array}$$ (4)

$$\begin{array}{cc} \langle {\left(y^{\left(i\right)}\right)}^{2}\rangle =1\; & \text{(unit}\;\text{variance),} \end{array}$$ (5)

$$\begin{array}{cc} \forall j\;s.t.\;j<i:{\left\langle y^{\left(i\right)}y^{\left(j\right)}\right\rangle }_{t}=0\; & \text{(decorrelation}\;\text{and}\;\text{order).} \end{array}$$ (6)

Eq (3) introduces the Δ-value which is a measure of the slowness of the signal *y*^(*i*)^(*t*). Eqs (4) and (5) are applied successively for increasing *i*. Eqs (4) and (5) avoid the trivial solution of a constant function, for which Δ = 0. Eq (6) ensures that SFA does not yield the same feature twice and that the extracted features are ordered according to the degree of their slowness.

The semantic network consists of converging layers of SFA nodes. Information is first extracted locally and then integrated into more global and abstract features, see [55] for a more detailed description. In each SFA node, the same processing steps are performed (Fig 1C, top right). The network was implemented using the MDP library [58]. It was trained sequentially from bottom to top on sequences of 10,000 images in each training session. Although SFA learns on sequences and the movement statistics determines which features are learned, SFA does not learn the movement statistics of the training sequences itself. In fact, the network learns to extract a semantic representation from a single input pattern, i.e., the extracted functions *g*^(*i*)^ operate on single input patterns. This makes SFA fundamentally different from low-pass filtering.

Due to our particular choice of the object’s movement parameters in the training sequences (mainly the translation and rotation speeds), the four slowest features that emerged from the trained SFA network are related to the coordinates of the object’s center and its orientation [59]. To illustrate the SFA output, we used input sequences where the object moves along a trajectory and rotates by 360 degrees (Fig 1D). We refer to the vector **y**_*i*,*t*_ of SFA features at a given time *t* in sequence *i* as the semantic, i.e., more abstract, representation of a single input image. After the semantic representation network had been trained, we used it to process sequences with different movement statistics. The temporal sequence of these semantic representation {**y**_*i*,1_,**y**_*i*,2_,**y**_*i*,3_, …}, describing the movement of the object in the input sequence *i*, is stored in the episodic memory system.

#### Sequence storage network

Sequences were stored in a simplified algorithmic model, in which they are not stored in their entirety, but as individual elements that are linked by “pointers”. The mechanism is illustrated in Fig 1E. Except for the last pattern of the sequence, each sequence element is associated with the next element in the sequence, which serves as the key for retrieving the next element. To perturb memory retrieval, we added noise $\epsilon_{t+1}^{\left(k\right)}∼N\left(0,\sigma_{n}^{2}\right)$ to the retrieval key $y_{i,t+1}^{*}$ to form the retrieval cue and then search for the stored element that is closest (according to the Euclidean distance) to the retrieval cue, i.e.,

$$\begin{array}{c} r^{\prime },s^{\prime }=\underset{r,s}{\text{argmin}}∥y_{i,t+1}^{*}+\epsilon_{t+1}-y_{r,s}∥. \end{array}$$ (7)

So, the retrieved pattern is

$$\begin{array}{c} y_{i,t+1}^{\prime }=y_{r^{\prime },s^{\prime }}, \end{array}$$ (8)

This retrieval process is then iterated with the next retrieval cue, which is the key associated with the last retrieved element. Either due to the retrieval noise or when two elements are identical, the retrieval process can return the wrong element.

To quantify the retrieval error, we calculated the Euclidean distance between the stored and retrieved sequence pattern by pattern:

$$\begin{array}{c} e\left(t\right)=∥y_{i,t}^{\prime }-y_{i,t}∥. \end{array}$$ (9)

In the simulation, we always store multiple sequences, with 50 elements each, in the network.

### Modeling the effect of adult neurogenesis in episodic memory storage

**Fig 2:**
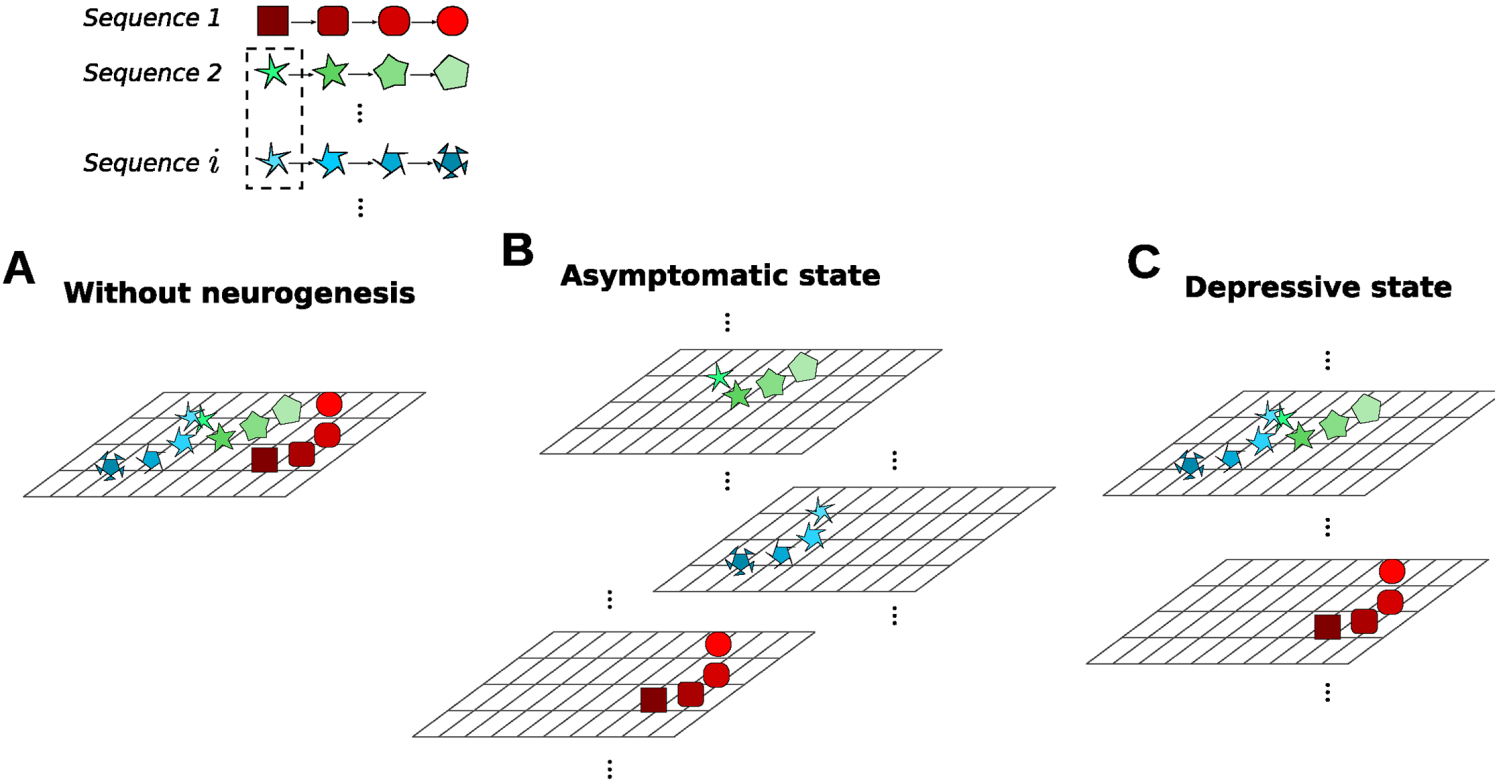
Illustration of the role of adult neurogenesis in the dentate gyrus. Top: Schematic of three stored sequences in the memory model, where the first elements in sequences 2 and *i* are similiar to each other. A: Without adult neurogenesis, the memory patterns are located in close proximity to each other in the memory space. B: In the asymptomatic state with a normal rate of adult neurogenesis, the augmentation with distinct pattern separation vectors distributes the sequences along an additional dimension in memory space. C: In the depressive state, the new sequence (*i*) is stored by re-using a pattern separation vector that had been assigned to a memory stored in a preceding asymptomatic state, based on the similarity of their first patterns. As a result, the two memory sequences, 2 and *i*, are more likely to interfere during retrieval.

We assume that AN affects episodic memory through pattern separation. In the following, we describe the abstract model that we use for pattern separation. A motivation in terms of neuronal mechanisms will be provided in the Discussion. In our model, the semantic representations **y**_*i*,*t*_ are driven entirely by the inputs and therefore reflect the correlations that are present in the inputs. Storing these correlated patterns would lead to interference between the patterns (Fig 2A). To avoid this interference, we augment every sequence element with an additional pattern separation vector **a**_*i*_, i.e.,

$$\begin{array}{c} \left[\begin{array}{c} y_{i,t}\\ a_{i} \end{array}\right] \end{array}$$

where $a_{i}^{\left(k\right)}∼N\left(0,\sigma_{a}^{2}\right)$ for all dimension *k*. In the higher dimensional space of augmented memory patterns, the patterns are more dissimilar to each other than the original patterns were (Fig 2B). We assume that a new pattern separation vector is generated for each new sequence that has to be stored in the network when AN occurs at a normal rate in DG. Each pattern separation vector **a**_*i*_ is associated with the first element of a sequence **y**_*i*,1_.

During retrieval, [yi,1ai] serves as the retrieval key for the next element in the sequence. The model searches for the augmented pattern that is closest to this retrieval key, plus retrieval noise. Note that retrieval noise is also added to **a**_*i*_. Since the pattern separation vector only plays an auxiliary role in memory storage and retrieval, retrieval performance is assessed based on the retrieved pattern $y_{i,t}^{\prime }$ alone, i.e., excluding the pattern separation vector (Eq 9).

### Modeling memory storage and retrieval in different disease states in MDD

**Fig 3:**
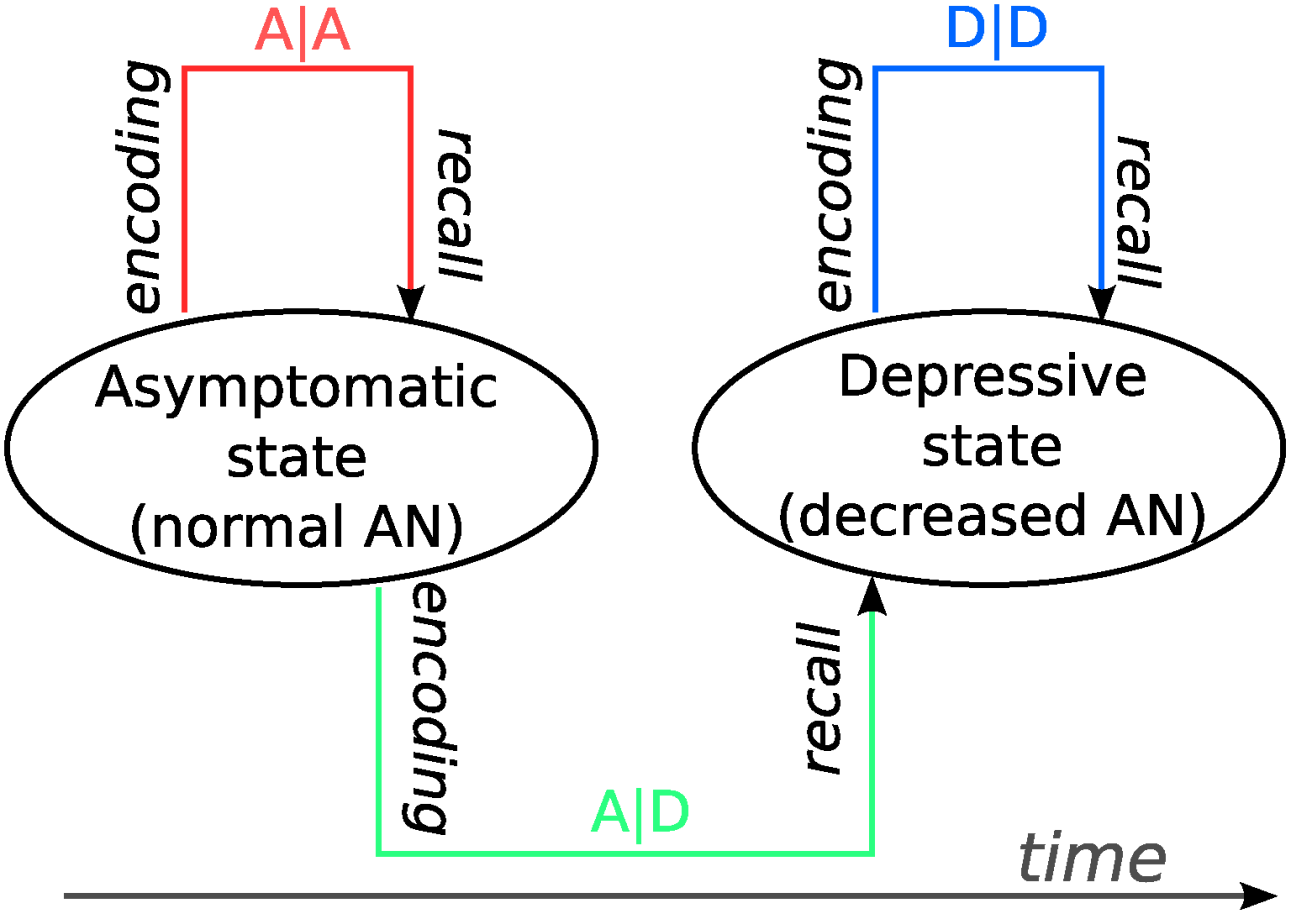
Three scenarios of memory storage and retrieval in the model. The rate of adult neurogenesis (AN) is normal in the asymptomatic state and reduced in the depressive state. The origin of the arrow indicates during which state the memory was stored, and the termination of the arrow indicates during which state the memory is retrieved. A|A: memories stored and retrieved in the asymptomatic state; A|D: memories stored in the asymptomatic state and retrieved in the depressive state; D|D: memories stored and retrieved in the depressive state.

In this study, we limit ourselves to considering only the asymptomatic state (A) and the depressive state (D). Based on the experimental evidence discussed above, we assume that the rate of AN is normal in the asymptomatic state and zero in the depressive state. The latter assumption implies that no new pattern separation vectors are generated for new sequences in the depressed state and previously generated ones are re-used. Four cases can be distinguished in principle based on which of the two states a memory sequence was stored and retrieved in (Fig 3). We use the notation “X|Y” to indicate that a memory was stored in state X and retrieved in state Y. The four possible cases are A|A, A|D, D|D, and D|A. We will only discuss the first three cases, because the D|A case can be decomposed into those memories for which A|A applies and those for which D|D applies. We return to this issue in the Discussion.

When memories are stored in the asymptomatic state, the pattern separation mechanism works just as described in the preceding subsection. However, a new mechanism is required when the pattern separation mechanism is used, but no new pattern separation vectors can be generated for new sequences, which is the case in the depressive state (Fig 2C). In this case, mature cells in DG that have already been associated with specific memories have to be reused when storing new memory sequences, i.e., the pattern separation vectors already in the system are reused. More precisely, for a new sequence **y**_*j*,⋅_, we determine the pattern separation vector **a**_*j*_ to be reused based on the similarity between the first patterns in the sequences, i.e., **y**_*j*,1_ is compared to **y**_*k*,1_ for all stored sequences *k* in A|D. So,

$$\begin{array}{c} i=\underset{k}{\text{argmin}}∥y_{j,1}-y_{k,1}∥, \end{array}$$ (10)

then

$$\begin{array}{c} a_{j}=a_{i}. \end{array}$$ (11)

Reusing the pattern separation vector **a**_*i*_ for the new sequence **y**_*j*,⋅_ introduces interference between the sequences *i* and *j* (Fig 2C, top).

## Results

### Pattern separation improves the robustness of memory retrieval

**Fig 4:**
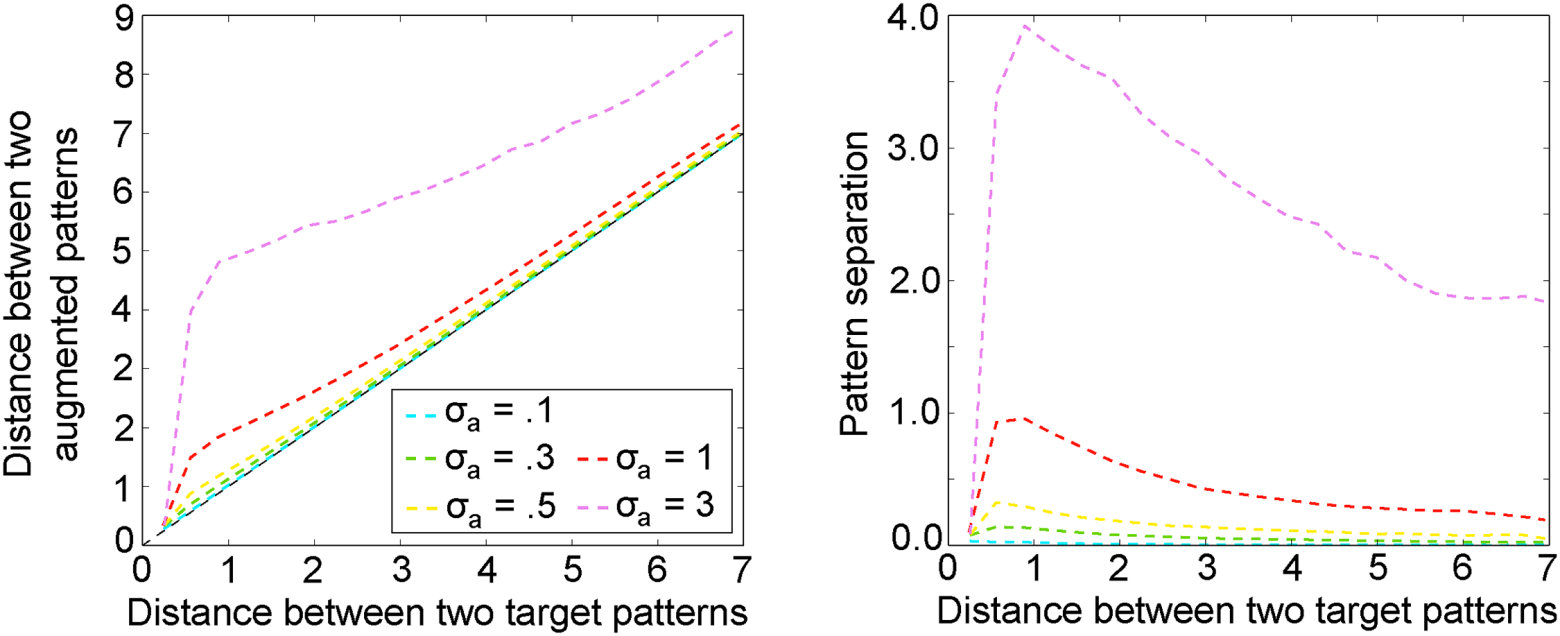
Augmentation with pattern separation vector leads to pattern separation. Left: The distance between pairs of augmented patterns (*D*_*a*_), i.e., containing the pattern separation vectors, against the distance between pairs of original patterns (*D*_*t*_). A curve above the diagonal means that the augmented vectors and more dissimilar than the original vectors, indicative of pattern separation. Right: Same data as left panel, but plotted to emphasize pattern separation (= *D*_*a*_ − *D*_*t*_).

We study the effect of augmenting memory patterns **y**_*i*,*t*_ with a pattern separation vector **a**_*i*_ on pattern separation in our model. Since the Euclidean distance between patterns plays an important role in retrieval in our model, we quantified the dissimilarity of patterns using the Euclidean distance. We find that distance between augmented patterns *D*_*a*_ are larger than the distance between the original target patterns *D*_*t*_ (Fig 4), indicating that pattern separation indeed occurs in our model. Furthermore, the effect of pattern separation is largest for highly similar patterns (*D*_*t*_ < 1) and for large variability in the pattern separation vector (large *σ*_*a*_).

**Fig 5:**
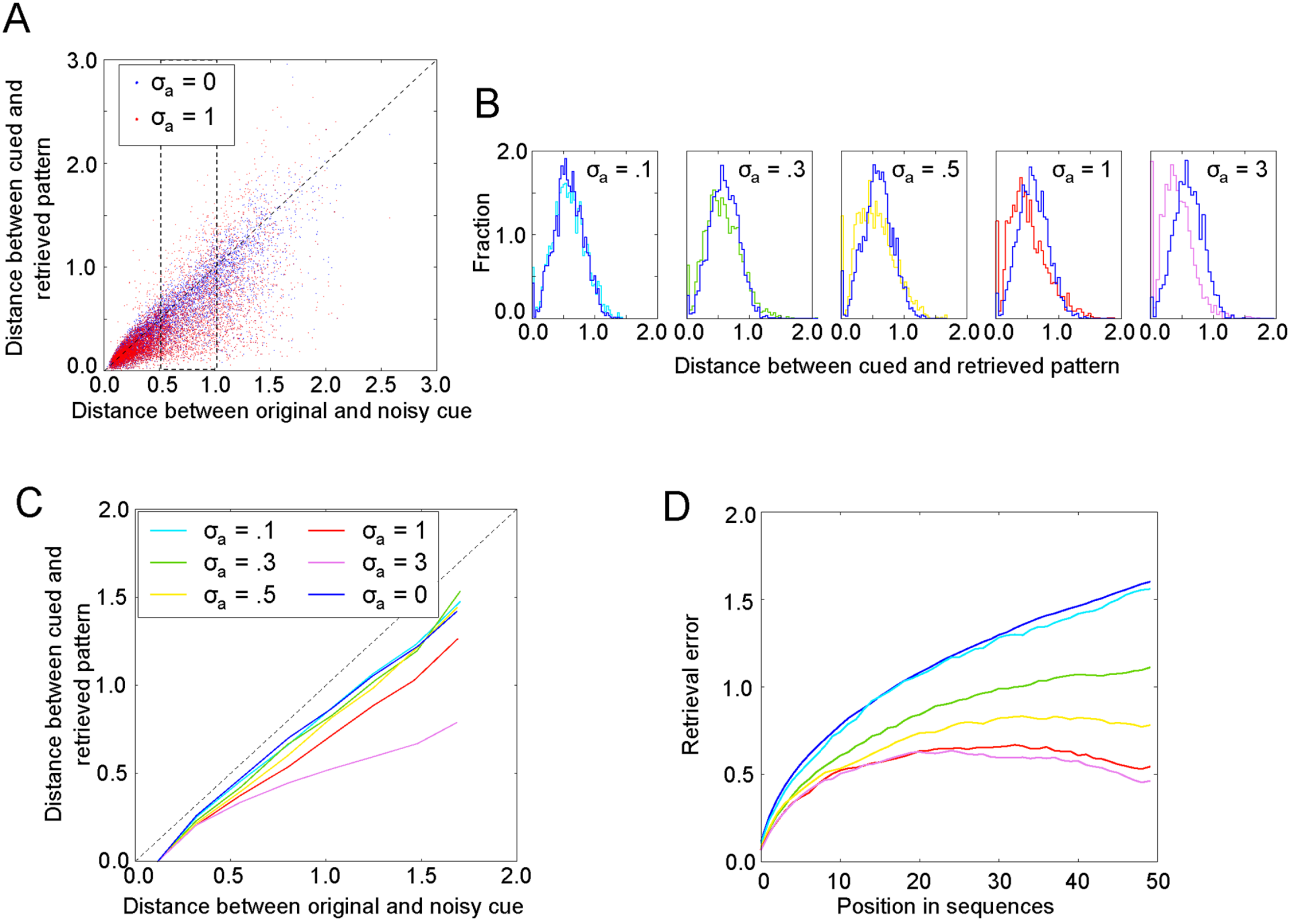
Pattern separation improves episodic memory retrieval. A: Example performance of single-pattern retrieval across different level of retrieval noise (raw data). B: Distribution of the distance between cued and retrieved patterns at different levels of pattern separation *σ*_*a*_ (only for the data within the dashed rectangle in A) as indicated by different colors. The legend is given in panel C, the reference *σ*_*a*_ = 0 is shown in dark blue. C: Average performance of single-pattern retrieval as a function of retrieval noise. D: Retrieval error for retrieval of sequences at different levels of *σ*_*a*_ (100 stored sequences, *σ*_*n*_ = 0.1, **a**: 2-D).

The next question is how pattern separation affects the retrieval performance in our model. We first tested retrieval of individual patterns. We randomly drew a stored pattern as the retrieval cue and performed a one-step retrieval. The retrieved pattern should be identical to the cue pattern with the retrieval noise removed. Fig 5A–5C shows the retrieval performance as a function of retrieval noise for different *σ*_*a*_. Note that the retrieval error represents only the distance between the originally stored **y**_*i*,*t*_ and the retrieved $y_{i,t}^{\prime }$ patterns (see Methods). The retrieval error curves lie below the diagonal for all *σ*_*a*_ we tested, even for *σ*_*a*_ = 0 (Fig 5C), which indicates that noise is reduced in the retrieval process. In other words, the model performs pattern completion. Furthermore, higher variability of the pattern separation vector leads to larger effects of pattern separation (Fig 5B and 5C). We will therefore use *σ*_*a*_ as a proxy for the degree of pattern separation. Our results confirm the common hypothesis that pattern separation make memory retrieval more robust.

Next, we analyzed how pattern separation affects the retrieval of memory sequences in a model that stored 100 sequences (random walk trajectory), each with an unique pattern separation vector. Consistent with the result for single pattern retrieval, introducing pattern separation into the memory network (0 ≤ *σ*_*a*_ ≤ 1), increases the retrieval accuracy of memory sequences (Fig 5D). However, large amounts of pattern separation (*σ*_*a*_ > 1) do not yield further improvement of the retrieval performance, indicating that pattern separation cannot fully eliminate the retrieval error in our model. In our model, DG AN is modelled by the generation of pattern separation vectors, which is parametrized by *σ*_*a*_. Better memory performance for (*σ*_*a*_ > 0) therefore means that AN improves episodic memory.

### Dynamics of memory retrieval in asymptomatic and depressive state

**Fig 6:**
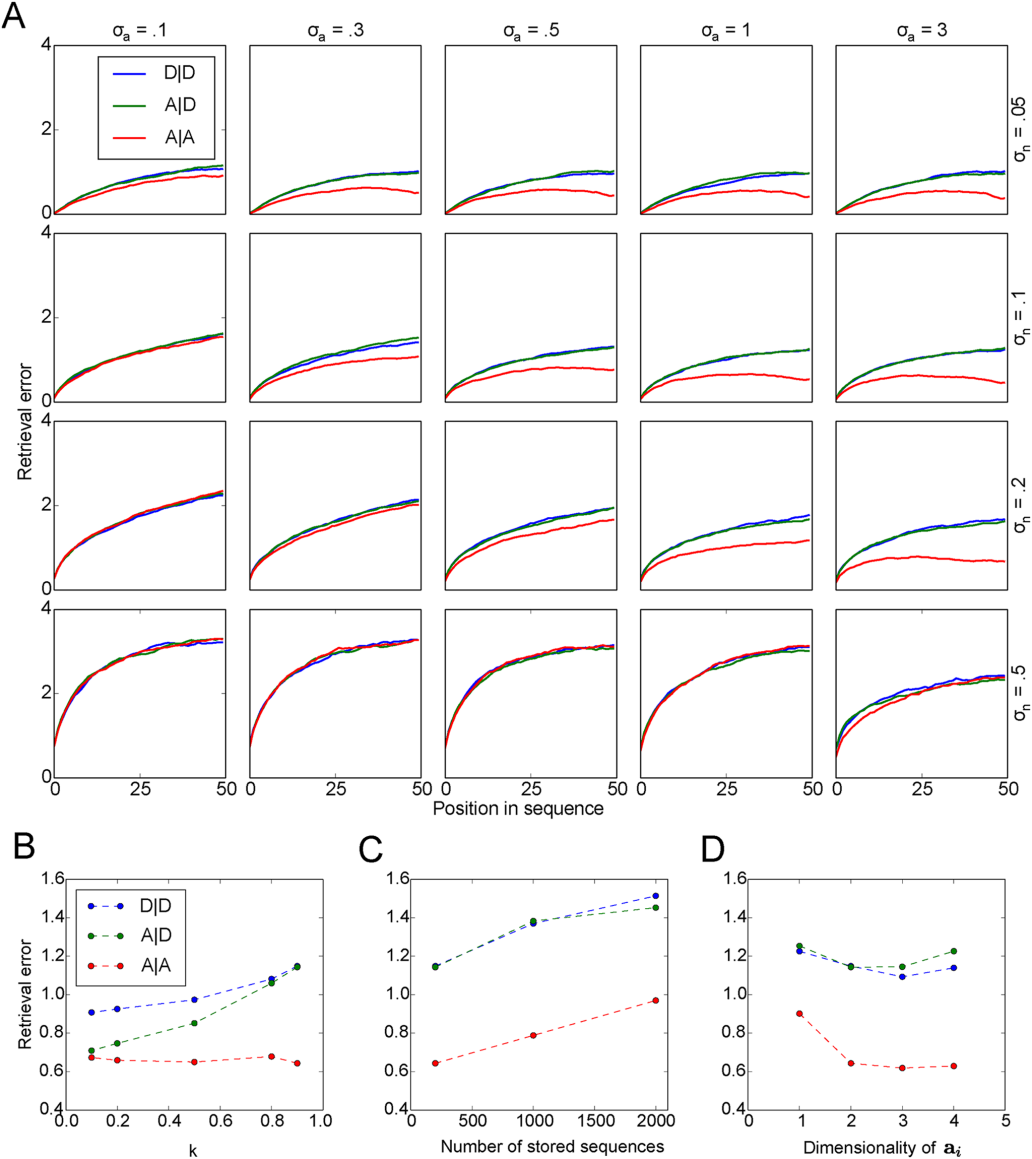
Sequential memory retrieval in asymptomatic and depressive states. A: Retrieval performance for the three cases A|A, A|D, D|D (indicated by color) at different levels of pattern separation *σ*_*a*_ (left to right) and retrieval noise *σ*_*n*_ (top to bottom). B: The duration of depressive episode affects the retrieval performance of A|D and D|D. Duration is measured by the fraction of memories stored in the depressive episode *k* (Eq 12). C: Increasing the number of stored sequences negatively impacts the retrieval performance in all cases, while the difference are preserved. D: Increasing the dimensionality of the pattern separation vector, up to a certain point, increases the difference between the A|A and the other cases. Values in B, C and D are calculated based on the 30^th^ element in the sequence (*σ*_*a*_ = 1, *σ*_*n*_ = 0.1). For A,B,C: **a**:2-D; for A,C,D: *k* = 0.9; for A,B,D: 200 stored sequences in both asymptomatic and depressive state respectively.

To test our hypothesis that a reduction of AN in DG induces pattern separation impairment, which in turn impairs episodic memory in depression, we study the retrieval quality of memories stored and retrieved in the asymptomatic and depressive states, respectively. Two hundred sequences are stored in each state. Specifically, we compared retrieval performance in the three cases: A|A, A|D and D|D for different levels of retrieval noise *σ*_*n*_ and pattern separation *σ*_*a*_. At low levels of pattern separation *σ*_*a*_ = 0.1, retrieval performance is comparable in the three cases (Fig 6A). Increasing the level of pattern separation (from left to right in Fig 6A), while keeping the level of retrieval noise fixed, improves the retrieval performance in all three cases, but the degree of improvement differs. When memories are stored and retrieved in the asymptomatic state (A|A), memory performance is better than if memories were retrieved in the depressive phase (A|D), or stored and retrieved in the depressive phase (D|D). This finding indicates that depression impairs memory performance. The higher the level of retrieval noise is, the more pattern separation is required to yield an advantage of A|A, or conversely an negative impact of depression. For example, for *σ*_*n*_ = 0.05, a difference is already apparent for *σ*_*a*_ ≥ 0.1, while for *σ*_*n*_ = 0.2, a difference is only apparent for *σ*_*a*_ ≥ 0.5. If retrieval noise dominates, i.e., *σ*_*n*_ ≥ 0.5, no amount of pattern separation yields a difference between A|A, A|D and D|D. We discuss these effects in more detail below. To rule out the probability that our results are specific to a particular input stimulus, we also studied the model with different objects (‘T’, ‘U’, ‘E’) as input stimuli and find very similar results (data not shown). Together, these results suggest that retrieval performance in our model depends on the mutual interaction between the retrieval noise and pattern separation and that a memory deficit in depression would not be expected in every case.

#### Impact of depressive episode duration on retrieval performance

fMRI studies suggest that in MDD the hippocampal volume is reduced progressively as the depressive episodes continues [60–62]. Since the hippocampus plays a critical role in episodic memory, one would expect that memory deficit worsen as depression lingers. We can study the effect of the duration of the depressive episode on memory performance in our model. Note our finding that across all the parameters shown in Fig 6A the performance in the case of A|D is always as impaired as in the case of D|D. This is somewhat puzzling since in the A|D case at least some sequences were stored in an asymptomatic phase, where the pattern separation vector was unique for each sequence. This result would be explained if new memory storage in the depressive phase leads to retrograde interference with previously stored memories. To test this hypothesis, we varied the relative duration of the depressive episode in our model, which means that a different relative number of memories are stored in the depressive and asymptomatic phases, respectively.

$$\begin{array}{c} k=\frac{N_{D|D}}{N_{A|D}+N_{D|D}}. \end{array}$$ (12)

The results in Fig 6A were obtained with *k* = 0.9. Across all values of *k*, we found that the retrieval performance in the case of A|A is the most accurate, while D|D is the worst. The difference between the two cases becomes more prominent for larger *k* (Fig 6B). We also find that for short duration of depression (small *k*), the retrieval performance of A|D is as good as the performance of A|A and then converges to the same level as D|D as the duration of the depressive episode increases (larger *k*). This indicates that even the remote memories formed in earlier asymptomatic state of the depressive patients are impaired as depression lingers.

#### The role of other model parameters

We studied the influence of two other parameters that have a potentially important role in memory performance in our model. First, we studied the role of the memory load by storing larger numbers of sequences in the memory network. Retrieval performance for all three cases becomes worse for higher memory load. The difference, however, between depressive state and asymptomatic state is almost constant (Fig 6C). Second, we expected the dimensionality of the pattern separation vector to influence pattern separation, i.e., higher dimensionality leads to larger pattern separation effects. Indeed, our results show that the advantage of the A|A case is already apparent with only a one dimensional pattern separation vector (Fig 6D). The effect is stronger for larger numbers of dimensions. However, for this particular set of memory sequences, increasing the dimensionality beyond two has little effect on memory performance in each of the three cases.

#### Accounting for the pattern of retrieval errors

Next, we explored how the difference in retrieval accuracy among the three cases arises. A retrieval error occurs when the retrieved pattern is different from the stored one, in other words, when retrieval jumps to an incorrect pattern. Intuitively, one might expect that the more frequently incorrect jumps occur, the larger the retrieval error is, but we found previously that the retrieval error is dominated by another process, namely the sequence divergence [55]. It refers to the tendency of two sequences that are close to each other at some point in time to diverge from each other over time. Since memory patterns are retrieved sequentially in our model, the movement along the sequence exacerbates the retrieval error, if the incorrect sequence diverges from the correct one. We therefore examined the sequence divergence as well as the probability of jumps to an incorrect pattern within the same sequences (*p*_*w*_) and between two sequences (*p*_*b*_) during retrieval. Sequence divergence is quantified by the increase in the distance between the subsequent elements of two sequences after two patterns in the respective sequences were the closest patterns to each other [55].

**Fig 7:**
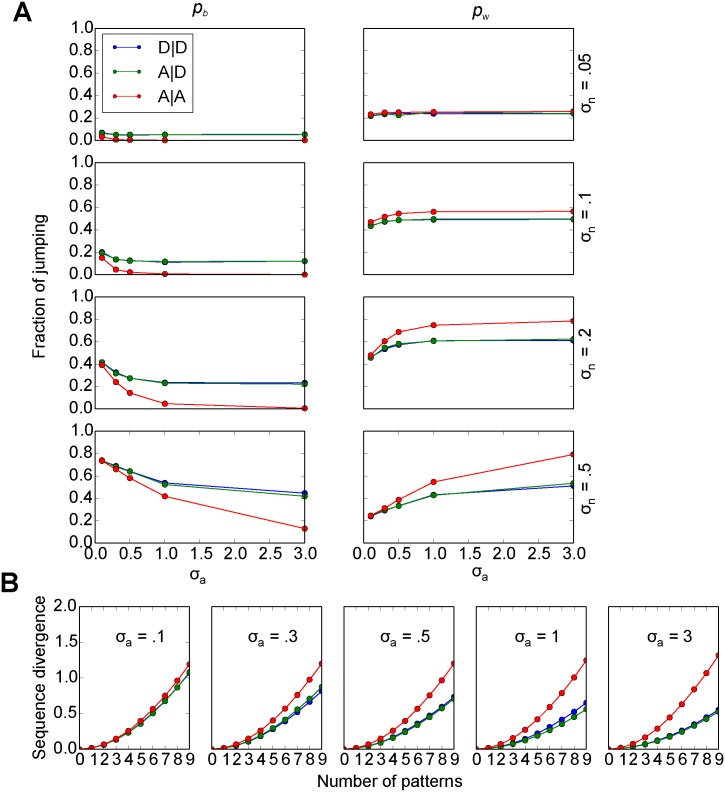
Probability of incorrect jumps and sequence divergence. A:left, probability of incorrect jumps between sequences (*p*_*b*_); right, probability of incorrect jumps within sequences (*p*_*w*_). B: Sequence divergence. For A, B: **a**:2-D, *k* = 0.2, 200 stored sequences.

Three observations account for the differences in retrieval error seen in Fig 6. First, increasing the retrieval noise leads to more faulty transitions both within and between sequences (Fig 7A, from top to bottom), which accounts for the increase in the retrieval error with increasing retrieval noise. Second, with the same level of retrieval noise, increasing the level of pattern separation (*σ*_*a*_) reduces the rate of faulty transitions between sequences, but increases the faulty transition rate within sequences. This is expected since pattern separation in our model acts to make sequences more distinct from each other. As a result, incorrect patterns within the same sequence are more often the closest element to the retrieval cue for the next element. This effect is more apparent in the A|A case than in the other two cases due to the stronger effect of pattern separation. Since jumps between sequences lead to larger errors than jumps within sequences, the differences in *p*_*b*_ between the three cases account for the differences in the respective retrieval errors (Fig 6A), except for the lack of a difference at high levels of retrieval noise (*σ*_*n*_ = 0.5).

The third observation fills this explanatory gap. Sequence divergence is maintained across different levels of pattern separation in the A|A case, while sequence divergence drops in the other two cases (Fig 7B). The latter effect is the result of reusing pattern separation vectors based on the similarity of the sequences in the A|D and D|D cases. Through this mechanism similar sequences become more clustered. Since pattern separation drives these clusters further apart, incorrect jumps go to similar sequences, thus reducing sequence divergence, when pattern separation is high. The lower sequence divergence offsets the higher jump probability *p*_*b*_ in the A|D and D|D cases and therefore reduces the difference to the A|A case in the retrieval error, but only if the jump probability *p*_*b*_ for the A|A case is not already close to zero. These conditions are satisfied for all levels of pattern separation, when *σ*_*n*_ = 0.5, which explains why the A|A case performs no better in this noise regime.

### Pattern separation at input stage

**Fig 8:**
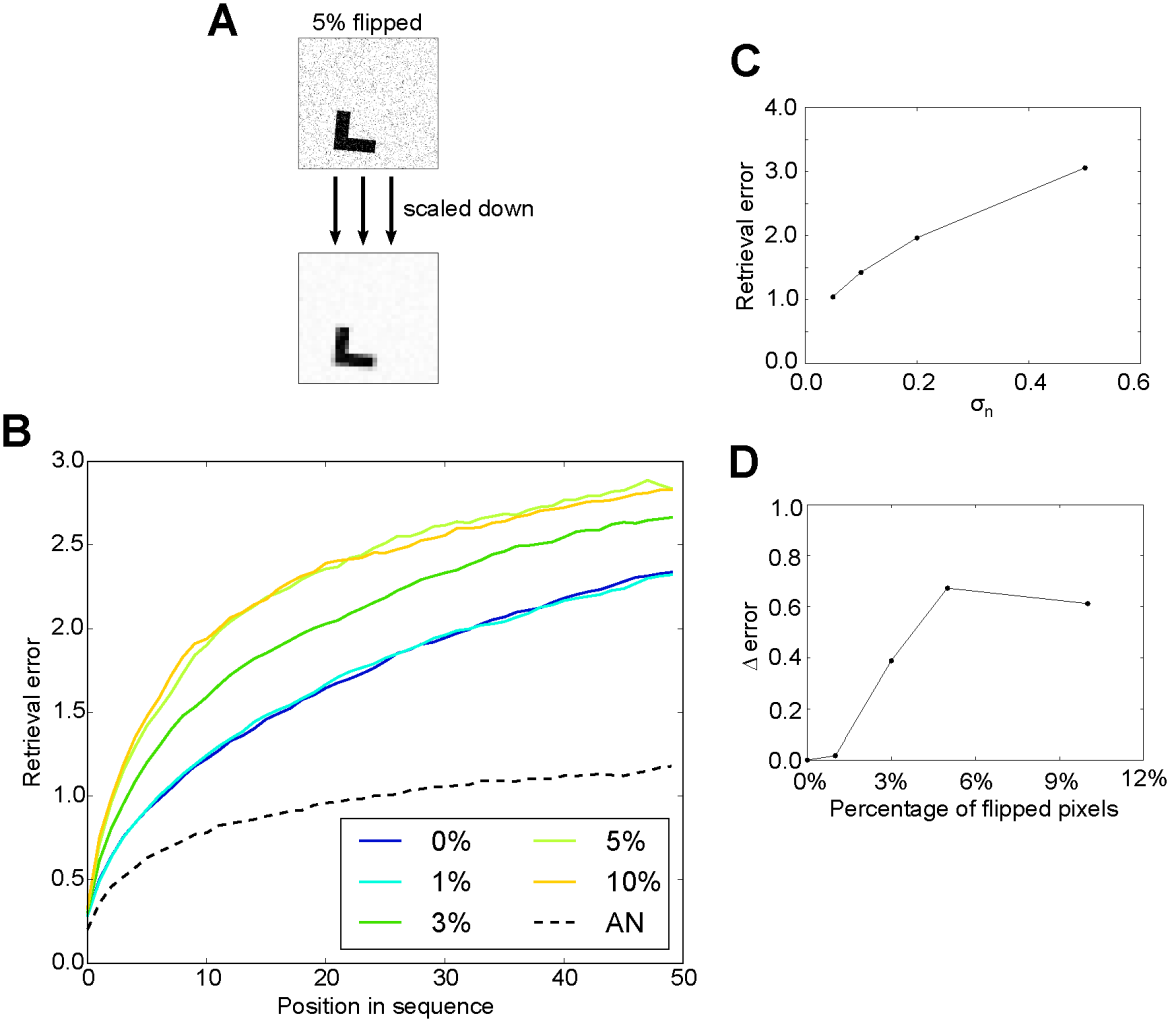
Retrieval performance with “pattern separation” in the sensory system. A: Example of the manipulated input patterns. Top: same pattern as in Fig 1B, but with 5% pixels flipped (300 × 300 pixels); Bottom: the scaled version. B: Retrieval error as a function of the fraction of randomly flipped pixels in the input image (*σ*_*n*_ = 0.2, 200 stored sequences). Dashed curve: retrieval performance of the model with neurogenesis (*σ*_*a*_ = 1) for comparison. C: With the same amount of noise in the input (1% flipped pixels), retrieval error increases monotonically with increasing retrieval noise *σ*_*n*_. D: The difference between the retrieval error for original patterns and that for noisy input pattern gradually increases with input noise (*σ*_*n*_ = 0.2). Values in C and D are drawn from the 30^th^ element in the sequence.

After showing that pattern separation improves episodic retrieval in our model, we asked whether pattern separation has to occur in the memory system or whether it could instead occur in the sensory system before the patterns are processed by the memory system. To study this question, we randomly flipped different numbers of pixels of each input image in the testing data (Fig 8A). No noise was added to the memory representations during storage. The way we added noise to the input patterns followed the same strategy that we used for pattern separation in the previous simulations. That is, the same pixels are flipped for all patterns within the same sequences, whereas different sets of pixels are flipped for the patterns in different sequences. Therefore, similar input patterns in different sequences should be separated. We tested whether this kind of pattern separation alleviates the interference between memories and facilitates the accuracy of memory retrieval.

Unlike what one might expect, we found that the retrieval performance is impaired by pattern separation in the sensory inputs (Fig 8B and 8D). Similar to previous results, retrieval noise impairs the retrieval performance (Fig 8C). These results indicate that pattern separation of sensory inputs does not necessarily mean the semantic representation patterns are separated as well, since the SFA network is performing a nonlinear operation. These results are consistent with our previous study [55], where we found that the episodic retrieval is more accurate when the semantic network is trained on the same image statistics that generates the inputs to be stored in memory, as compared to when the image statistics differ. Episodic retrieval is impaired when we add noise to the input, because by doing so we changed the input statistics after the semantic network had been trained. We therefore conclude that pattern separation at the sensory stage is not effective and therefore has to occur in the memory system.

## Discussion

We have developed a computational model to study episodic memory deficits in MDD. We assumed that MDD is associated with a reduction of AN in the DG, and that this reduction in AN impairs pattern separation. We hypothesized that the impairment of pattern separation in turn reduces the accuracy of episodic memory retrieval. In our model, episodic memories are encoded based on a semantic representation of the sensory inputs [55]. We investigated episodic memory deficit in MDD with an intact semantic system, which is consistent with observations that semantic memory is not affected in MDD [11, 12]. Our model of episodic memory is built around the idea that episodic memories are best represented by sequences of neural activity patterns [28, 29, 56, 63]. This aspect distinguishes our model from other models of neurogenesis, which only consider the storage and retrieval of static patterns.

### Correspondence to neuronal mechanisms underlying pattern separation

Even though our model does not reflect the anatomy and physiology of the hippocampus, it nevertheless describes hippocampal function at an abstract level and the functions of our abstract model can be roughly mapped onto the hippocamal circuit. The hippocampus has been found to be essential for sequence memory [64, 65] and we previously hypothesized that the hippocampal circuit is optimized for storing sequences of neural activity patterns [28]. During episodic memory storage, input patterns are mapped onto pre-existing intrinsic sequences of neural activity in CA3. In CA3, sequences are thought to be generated by the dynamics of its recurrent network, e.g. [66]. A given state of the network drives the next state through the recurrent synapses. In our model, we approximate this process in our sequence retrieval network, where the sequence elements are linked by associating each element with a retrieval key for the next element. Thus, the sequence elements **y**_*i*,*t*_ correspond to activity patterns in CA3.

The mechanism that we propose for pattern separation also has a correspondence in the hippocampal circuitry. Since newborn neurons are newly integrated into the network and are more excitable [67, 68], AN allows the DG to generate uncorrelated patterns of activity when new inputs arrive via EC. We thus propose that the pattern separation vector **a**_*i*_ reflects the activity patterns in DG. The augmented patterns [yi,tai] therefore represents the joint space of CA3 and DG patterns (Fig 2B). Consequently, memory retrieval based on the augmented pattern in our model corresponds to a bidirectional interaction between DG and CA3 during retrieval [69]. The choice in our model to retrieve the pattern separation vector **a**_*i*_ based on the initial retrieval cue **y**_*i*,1_ is motivated by the fact that the same EC input drives CA3 and DG activity and that the feedforward connections EC-CA3 and EC-DG can associate the input pattern with the activity patterns in the target areas [70]. Note that our suggestion that DG is involved in memory retrieval is a novel prediction. Other authors have previously concluded that DG is only important during memory formation, but not during retrieval [31, 71]. Finally, our choice to determine retrieval performance based only on the original component of the pattern $y_{i,\cdot }^{\prime }$ is well-justified because CA3 directly projects to output structures downstream, while DG does not.

In our model, the pattern separation vector is identical for all patterns within the same sequence, while different pattern separation vectors are generated for different sequences. This assumption is consistent with the temporal tagging hypothesis [51]. It was proposed that memories formed at distinct times would be represented by different groups of neurons in DG since newborn cells continue to be integrated into the network. As a result, memories formed close in time would be associated by the same group of immature DG granule cells (pattern integration), while memories formed at times far apart would be represented by distinct sets of DG neurons. Similarly, the functional cluster hypothesis proposes that the same contexts are represented by DG cells that were born simultaneously [43]. We therefore conclude that our abstract model is firmly rooted in the neuronal mechanisms underlying pattern separation in the hippocampal formation.

### Rate of adult neurogenesis and memory persistence

Empirical evidence suggests that increases in the rate of AN improves the performance on a variety of memory tasks [24, 41]. Here, we find that increasing AN up to a certain level improves memory performance (Figs 5 and 6). Moreover, since retrieval performance in our model depends on the interaction between the retrieval noise and pattern separation, memory deficits would not be expected in every case of MDD. Indeed, some studies failed to find episodic memory deficits in depressed individuals [72, 73]. We hypothesize that the retrieval error in our model is determined by task demands, the subject’s level of engagement, and neural processing. Pattern separation would be affected by the rate of DG AN, and the severity, and perhaps the duration, of the depressive phase. To test these predictions, future experimental studies could systematically vary the rate of AN and retrieval noise, and measure the affect of these manipulations on retrieval performance.

What is currently missing from our model is a detrimental effect of AN on memory. Experimental [47, 74] and computational [47, 75] studies have found that a high rate of AN leads to faster forgetting. Apparently, integrating new neurons into the hippocampal circuit affects memories that are already stored, because new cells and new connections compete with existing ones. In other words, there is trade-off between plasticity and stability.

### Episodic memory deficits in MDD

Our model predicts that MDD has an retrograde effect on episodic memory retrieval (Fig 6B). That is, memories retrieved in a depressive state are less accurate, even if they had been stored in a preceding asymptomatic state (A|D), as compared to memories that were stored and retrieved in an asymptomatic state (A|A). Studies of auto-biographical memories, which we discuss below, appear to support a retrograde effect of MDD on previously formed memories. However, to the best of our knowledge, a retrograde effect has yet to be demonstrated under laboratory-controlled conditions. Moreover, we find that memory deficits depend on the duration of the depressive episode. The longer the depressive episode lasts, the more severe the memory performance becomes.

In addition to the three case discussed in our study (A|A, A|D, D|D), there is another possible scenario. A memory can be stored in the depressive state and retrieved in the asymptomatic state (D|A). While this case is distinct from the other three, we did not include it in our study because it can be viewed as a composite of two other cases. Memories stored in the depressive state are not assigned a distinct pattern separation vector, while memories stored in the subsequent asymptomatic state are. New memories would therefore rarely interfere with previously stored memories and the D|A case can be decomposed into those memories that fall under the A|A case (new memories) and those under the D|D case (old memories). Our model, therefore, predicts that the memory deficit is not rescued when the depressive state ends. In other words, the damage caused in the depressive state by interference in the memory system cannot be undone. By contrast, the A|D case cannot be decomposed, because the pattern separation vector generated during the asymptomatic phase are re-used during the depressive phase, which leads to retrograde interference.

We found that the type of error committed during memory retrieval differs during MDD (Fig 7). According to our model during MDD patients might more frequently confuse memories formed at different timepoints than healthy controls. Somewhat paradoxically, it also predicts that controls incorrectly report events that occurred close in time more frequently than patients do. This novel prediction awaits testing in experimental studies.

### Shifting from episodic to semantic memory in MDD

Apart from impairments in episodic memory, patients suffering from MDD also show over-general memories [12, 76–78]. When subjects are asked to recall a particular event from their personal history related to a given cue, patients, more often than controls, retrieve rather general information that summarizes a category of events [12, 77]. This is called the over-general memory effect. For instance, when cued with “enjoy” to recall an event, patients tend to produce generic answers, e.g., “I enjoy a good party”, whereas controls produce specific memories such as “I enjoyed Jane’s party last Saturday”. To account for this effect, Williams et al. [77] adopted the Conway and Pleydell-Pearce model [79], which suggests that autobiographical memories are arranged in a hierarchical structure with the general categories at the top, specific categories in the middle and specific event memories at the bottom. Autobiographical memories are retrieved by traversing this memory structure from top-to-bottom. Williams et al. suggest that MDD patients block the access to specific event memories in order to avoid retrieving painful memories and therefore end the retrieval process at an abstract level.

By contrast, we propose that the same episodic memory deficit that we studied here might be sufficient to account for over-general memories, too. Episodic memories together with personal semantic information forms autobiographical memory. Episodic memories are about specific events, whereas semantic memories refer to general facts. Therefore, over-general memory can be seen as a shift from the retrieval of episodic memories to the retrieval of semantic memories. If episodic memory retrieval is impaired during MDD, retrieval of autobiographical memories is more likely to result in a semantic memory which is mostly preserved during MDD. This shift from a reliance on episoidic memory to reliance on semantic memory appears as a shift from specific to over-general memories. This account is consistent with a previous suggestion that over-general memory could result from reduced episodic recall, increased semantic recall or the combination of both [12].

In conclusion, the model we present here might be able to account for both over-general memories and episodic memory deficits in MDD.

## Supporting information

S1 File Python code. (PDF)
