# Supplementary material for: The reduction of adult neurogenesis in depression impairs the retrieval of new as well as remote episodic memory
Source: PLoS One. 2018 Jun 7;13(6):e0198406. doi: 10.1371/journal.pone.0198406 (PMC5991644; doi:10.1371/journal.pone.0198406)
Supplement: S1 File — (PDF) [file pone.0198406.s001.pdf]

Supporting information

All code for this manuscript is fully available online at
https://github.com/inkfang/Episodic-memory-deficits-in-depression .
